# Severe Dyspnea Is an Independent Predictor of Readmission or Death in COPD Patients Surviving Acute Hypercapnic Respiratory Failure in the ICU

**DOI:** 10.3389/fmed.2018.00163

**Published:** 2018-05-29

**Authors:** Elise Dupuis-Lozeron, Paola M. Soccal, Jean-Paul Janssens, Thomas Similowski, Dan Adler

**Affiliations:** ^1^Division of Clinical Epidemiology, Geneva University Hospitals, Geneva, Switzerland; ^2^Division of Pulmonary Diseases, Geneva University Hospitals, Geneva, Switzerland; ^3^Department of Internal Medicine Specialties, Geneva Medical School, Geneva, Switzerland; ^4^Sorbonne Universités, UPMC Université Paris 06, Institut National de la Santé et de la Recherche Médicale, UMRS1158 Neurophysiologie Respiratoire Expérimentale et Clinique, Paris, France; ^5^AP-HP, Groupe Hospitalier Pitié-Salpêtrière Charles Foix, Service de Pneumologie et Réanimation Médicale (Département “R3S”), Paris, France

**Keywords:** COPD, dyspnea, ICU, readmission, death

## Abstract

**Background:** Predicting outcome after index admission in the ICU for COPD-related acute hypercapnic respiratory failure (AHRF) is difficult. Simple tools to stratify this risk and to promote interventions to mitigate it are needed.

**Aim:** To prospectively evaluate the ability of severe dyspnea (NYHAIII-IV) to predict hospital readmission or death in COPD patients surviving AHRF in the ICU.

**Methods:** 50 consecutive COPD patients were recruited from a larger cohort of patients (*n* = 78) surviving AHRF in the ICU. All predictive variables were collected within 15 days after resolution of respiratory failure before hospital discharge. COPD was diagnosed by spirometry. Heart failure was diagnosed with clinical rules and echocardiography. NYHA was measured upon hospital discharge. Hospital readmission and death were recorded at regular intervals for 3 months.

**Results:** 21/50 (42%) COPD patients died or were readmitted to the hospital during the observation period: 12 out of the 20 NYHA III-IV patients (60%) and 8 out of the 28 NYHA I-II patients (29%). NYHA III-IV was associated with risk of readmission or death (univariate HR: 2.73, IC95: 1.11–6.69, *p* = 0.028). After controlling for age, FEV1, heart failure and BMI, NYHA III-IV remained associated with readmission or death (multivariate HR: 2.71, IC95: 1.06–6.93, *p* = 0.038).

**Conclusions:** Our findings suggest that severe dyspnea measured upon hospital discharge in COPD patients surviving AHRF can stratify patient's risk of 3-month readmission or death.

## Introduction

Patients with chronic obstructive pulmonary disease (COPD) who survive acute hypercapnic respiratory failure (AHRF) treated with non-invasive ventilation (NIV) in the intensive care unit (ICU) have a high risk of short-term readmission and death (1). Outcome after an index hospital admission can be estimated by multidimensional scores combining pulmonary function, the history of past exacerbations (2), and comorbidities (3), but these scores have never been tested in severe COPD patients surviving ICU. In this setting, where performing cardiorespiratory workups is often difficult and where informing patients and their families is a major stake (4), simple tools to assess short-term outcome are urgently needed. Dyspnea is associated with poor outcome in the general population, most probably as a proxy for cardiac and respiratory diseases (5). It is also recognized as an independent predictor of survival in COPD with a stronger relationship to mortality than that observed for forced expiratory volume in 1 s (FEV1) (6). We hypothesized that the presence of severe dyspnea measured prospectively upon hospital discharge would be predictive of hospital readmission or death after an index admission in the ICU for COPD-related AHRF.

## Methods

### Patients

During the study period (2012–2014), 78 consecutive patients surviving AHRF mimicking COPD exacerbation and treated by NIV in the ICU were screened. AHRF was confirmed by a pH <7.35 with an arterial carbon dioxide tension (PaCO_2_) of >6.5 kPa and the need for NIV in the ICU.

### Data collection

The following were collected within 15 days after ICU discharge before hospital discharge: demographic and anthropometric data, pulmonary function tests and transthoracic echocardiography using standardized protocols. NYHA class was assessed upon hospital discharge. Diagnosis of COPD was confirmed in all patients by the presence of post-bronchodilator fixed ratio FEV_1_/FVC < 0.7. Heart failure was diagnosed after careful history and physical examination in addition to evidence of systolic (LVEF < 55%) or diastolic dysfunction (7). Hospital readmission and death were recorded at regular intervals for 3 months by reviewing medical records or through telephone calls to the patients or to family members.

All included patients signed written informed consent forms. The study protocol was approved by the University of Geneva institutional review board (#11–238).

### Statistical analysis

Data are expressed as median (interquartile range, IQR). We constructed a Kaplan-Meier cumulative-event curve for hospital-free survival. The data were censored at the 2nd follow-up visit, at least 3 months after ICU discharge. Log-rank test was used to compare the curves in two groups (categorical classification of dyspnea NYHA I-II versus NYHA III-IV). Unadjusted and adjusted Cox proportional hazard models were used to test the effects of NYHA, age, BMI, FEV1% of predicted, and heart failure on outcome. Variables that did not satisfy the proportional hazard assumption were categorized if needed and the multivariate Cox model was stratified on them. We report log-rank test for those variables. A p value of <0.05 was considered statistically significant. Hazard ratios (HRs) are given with 95% confidence intervals (95%CIs). All analyses were performed using R version 3.3.0 (R core Team, 2016).

## Results

**Figure 1:**
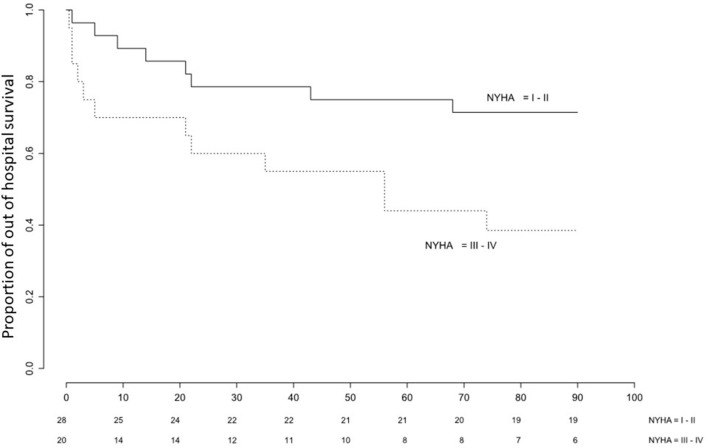
Kaplan-Meier plot of time to event (readmission or death) in COPD patients surviving index ICU admission for acute hypercapnic respiratory failure stratified on NYHA class (III-IV vs. I-II).

Of the 78 consecutive AHRF patients screened, 50 were diagnosed with COPD. Twenty-eight were NYHA class I or II, 20 were NYHA class III-IV, and NYHA class was missing in two cases (Table 1). Follow-up was complete for all patients and median follow-up was 90 days. 21 patients (42%) reached the combined endpoint of readmission or death during the study period (12 of the 20 NYHA III-IV patients [60.0%, 95% confidence interval [CI]: 36.0–80.9]; 8 of the 28 NYHA I-II patients [28.6%, 95%CI: 13.2–48.7]). Of those, 6 deaths (12%) were recorded during the 3 month observation period (4 of the 20 NYHA III-IV patients, respectively 2 of the 28 NYHA I-II patients). The probability of event-free survival was lower in NYHA III-IV patients compared to NYHA I-II (Figure 1, *p* = 0.023 on log-rank test). Table 2 shows univariate and multivariate Cox regression models regarding combined endpoint of readmission or death. In a model controlling for age, heart failure, BMI and stratified on FEV1, NYHA III-IV remained independently associated with readmission or death (HR: 2.71; 95% CI: 1.06–6.93; *p* = 0.038).

**Table 1:** Baseline Characteristics of the study population^*^ according to categorical classification of Dyspnea (NYHA I-II vs. NYHA III-IV).

**Patients Characteristics**	**NYHA I-II (*n* = 28)**	**NYHA III-IV (*n* = 20)**	***P*-Value**
Age, years (IQR)	66 (60–75)	70 (67–77)	0.193
Gender, male (%)	19 (68)	9 (45)	0.144
Current smoker (%)	19(68)	9(45)	0.353
BMI, kg/m2 (IQR)	26.5 (21.5–30.8)	31.1 (26.5–40.5)	0.060
Heart failure (%)	13 (46)	14 (70)	0.152
FEV1, % of predicted (IQR)	47.5 (32.8–53.3)	43.5 (34.8–52.3)	0.730
Past hospital admission during previous yr-any vs. none(%)	7(25)	3(15)	0.481
SAPSII, IQR	36.5 (26.8–49.5)	37 (29–43.3)	0.892
Length of stay in ICU, days (IQR)	2.5 (1.8–4)	3 (1.8–5)	0.815
PAP treatment on hospital discharge (NIV or CPAP) (%)	9(32)	11(55)	0.232

NYHA, New York Heart Association; IQR, interquartile range; BMI, body mass index; FEV1, forced expiratory volume at 1 s; SAPS, Simplified Acute Physiology Score; ICU, intensive care unit.

* *50 COPD patients were identified, but the NYHA class information missed in two cases*.

**Table 2:** Risk Factors for Adverse Outcome Following ICU discharge tested in Cox univariate and Cox multivariate Models.

	**Univariate HR (95%CI)**	**p value**	**Multivariate HR (95%CI)**	***p*-value**
NYHA III-IV	2.73 (1.11–6.69)	0.028	2.71 (1.06–6.93)	0.038
Past hospital admission during previous yr-any vs. none	1.23 (0.45–3.39)	0.690	–	–
PAP treatment on hospital discharge vs. no	1.57(0.65–3.78)	0.311	–	–
Age (per 10 years increase)	1.50 (0.92–2.45)	0.1	1.32 (0.75–2.32)	0.332
Heart Failure	1.19 (0.48–2.91)	0.708	1.01 (0.30–3.32)	0.992
BMI (per 1 kg/m2 increase)	0.99 (0.94–1.04)	0.716	1.00 (0.94–1.06)	0.916
FEV1% of predicted^**^	*P*-value for log-rank test = 0.501			

ICU, intensive care unit: HR, hazard ratio; NYHA, New York Heart Association; BMI, body mass index

** *FEV1: forced expiratory volume at 1 s categorized as follows: FEV1 <30% of predicted, FEV1 ≥ 30% and <50% of predicted, FEV1 ≥ 50% of predicted*.

## Discussion

Current evidence suggests that index ICU admission often marks a turning point in the course of COPD with high readmission rate and increased mortality. Yet, patients and their caregivers are poorly informed about its prognostic importance (4). This is partly due to the challenge in identifying predictive factors in the ICU setting where the acutely altered condition of the patients makes spirometric and exercise assessment difficult and of questionable value. Our data indicate that dyspnea, when rated early after ICU discharge on the NYHA scale, is independently associated with poor outcome even after adjustment for four major clinical features commonly associated with dyspnea, i.e. age, BMI, the severity of airflow obstruction (FEV1), and the presence of heart failure. Of note, in our study, neither past hospital admission in the previous year, nor treatment with home positive airway pressure on hospital discharge were associated with readmission or death. This does not mean that they are not predictors of outcome in COPD (8, 9), but rather suggests that stronger factors are involved. NYHA, which is both a marker of dyspnea and frailty (10) could be a candidate factor in patients with physical deconditioning worsened by the acute exacerbation and the corresponding bed rest in ICU (11). This hypothesis is in line with current evidence that post-exacerbation respiratory rehabilitation (a comprehensive and multidisciplinary therapeutic intervention) reduces dyspnea, use of healthcare resources, readmissions, and mortality (12).

Our study has inherent limitations related to its observational design and to a limited sample size. We acknowledge that our results cannot be generalized to stable COPD patients treated in the outpatient clinic or even to COPD patients with severe exacerbation admitted in the general ward. In such cases, validated multidimensional indexes such as BODE (13), BODEx (2), or CODEX (3) are available. The strength of our finding lies in its simplicity.

Although interventional studies are needed, we suggest that the allocation of a COPD patient to NYHA dyspnea class III or IV on hospital discharge after AHRF should be an incentive to implement post-discharge comprehensive COPD care to reduce readmissions (14). These include early rehabilitation that may also provide the right setting to engage a discussion on patient's personal values and preferences, e.g., regarding advance care planning. To our knowledge, no other intervention is of proven efficacy to reduce early readmission and mortality. On the basis of our results, we encourage clinicians to systematically evaluate dyspnea upon hospital discharge in COPD patients recovering from AHRF.

## Author contributions

ED–L, PS, J-PJ, TS, and DA designed the study. DA coordinated the study. DA responsible for patient screening, enrollment, and follow-up. ED-L and DA performed statistical analysis. ED-L, PS, J-PJ, TS, and DA analyzed the data and wrote the manuscript.

### Conflict of interest statement

The authors declare that the research was conducted in the absence of any commercial or financial relationships that could be construed as a potential conflict of interest.
